# Therapeutic promise and challenges of targeting DLL4/NOTCH1

**DOI:** 10.1186/2045-824X-3-17

**Published:** 2011-08-08

**Authors:** Minhong Yan

**Affiliations:** 1 Department of Molecular Biology, Genentech, Inc. South San Francisco, CA 94080, USA

## Abstract

DLL4-mediated NOTCH1 signaling represents an essential pathway for vascular development and has emerged as an attractive target for angiogenesis-based cancer therapies. However, newly reported toxicity findings raise safety concerns of chronic pathway blockade. Lessons learned from the development of γ-secretase inhibitors (GSIs) might offer insights into how to safely harness this important signaling pathway.

## DLL4/NOTCH1 signaling pathway in angiogenesis

In metazoans, the evolutionarily conserved NOTCH pathway functions as an essential mechanism to regulate numerous cell fate/lineage decisions during embryogenesis, postnatal development, and in the maintenance of adult tissue homeostasis. NOTCH receptors are normally constrained in a dormant state. Ligand binding exposes the ADAM protease cleavage site that is normally buried within the negative regulatory region (NRR)[1]. Subsequent intramembrane cleavage catalyzed by γ-secretase, a multisubunit protein complex, permits the release of the intracellular portion (NICD) from the cell membrane and its entry into the nucleus where it forms a transcriptional activation complex. In mammals, the NOTCH signaling apparatus consists of four single-pass transmembrane receptors (NOTCH1-4) and at least five membrane-anchored ligands (Jagged1, 2 and Delta-like or DLL1, 3 and 4). Despite the apparent redundancy of multiple NOTCH ligands and receptors expressed in the vascular system, recent studies have revealed that the DLL4-NOTCH 1 interaction appears to be the dominant functioning component in the vascular system. DLL4 was initially identified as an endothelium-specific NOTCH ligand [2-5]. Haploinsufficiency of *Dll*4 in mice results in early embryonic lethality due to severe vascular defects including impaired arteriogenesis, disrupted vascular hierarchy, and enhanced vascular density with reduced vessel caliber [2,6,7]. This uniquely non-redundant role of DLL4 goes beyond early embryonic development. Studies utilizing a DLL4-selective antagonistic antibody demonstrate that DLL4 is also essential for early postnatal vascular development, angiogenesis during pathological wound healing (unpublished observations) and tumor angiogenesis [8]. Compared to DLL4, NOTCH1 is more broadly expressed and targeted disruption of *Notch1 *results in vascular defects similar to what has been observed with *Dll4 *deficiency [9,10]. Moreover, results from recent work using paralogue-specific antibodies indicate that NOTCH1 inhibition alone is sufficient to disrupt angiogenesis [11].

Studies in multiple model systems have revealed important insights into the function of Dll4/NOTCH1 signaling in angiogenesis and the underlying mechanism of vascular defects resulting from attenuated DLL4/NOTCH1 activity [11,12]. Excessive angiogenic sprouting, branching and increased endothelial cell proliferation are commonly associated with blockade of DLL4/NOTCH 1 signaling. Signaling induced by DLL4 through NOTCH1 causes downregulation of VEGFR2, whereas blocking DLL4 signaling leads to increased expression of VEGFR2 and VEGFR3 [13]. Therefore, DLL4/NOTCH1 signaling is apparently required to restrain the magnitude of response of endothelial cells to angiogenic stimuli. The chaotic angiogenesis resulting from DLL4/NOTCH1 blockade may reflect unrestricted VEGF signaling and disruption of a dynamic balance between tip cells and stalk cells during angiogenic sprouting [14].

## Therapeutic promise

The initial speculation of DLL4 signaling involvement in tumor angiogenesis came from expression analyses of DLL4 [12]. Results from a flurry of recent studies confirmed that targeting DLL4/NOTCH1 signaling has a profound impact on tumor angiogenesis and growth. Pathway blockade was achieved using either a DLL4-selective neutralizing antibody [8,15,16], a NOTCH1-selective antagonistic antibody[11], a soluble DLL4 fusion protein[17,18], or a soluble NOTCH fusion protein[19]. These reagents demonstrated broad and robust anti-tumor activity in a wide variety of tumor models.

**Figure 1:**
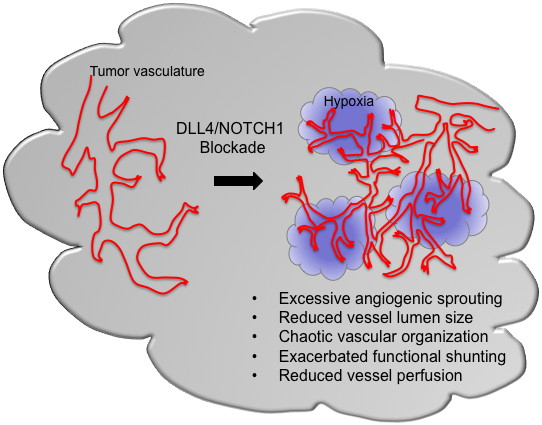
**Potential effects of DLL4/NOTCH1blockade on tumor vasculature**.

While most of the current anti-angiogensis approaches act through a reduction or elimination of tumor blood vessels, DLL4 blockade results in the formation of a non-functional vasculature incapable of supporting tumor growth (Figure 1). Histological analysis of tumors treated with DLL4/NOTCH1 inhibitors has revealed that the reduced tumor growth is associated with an apparent increase in tumor vascular density. However, labeling with an intravascular tracer has demonstrated these vascular structures to be poorly perfused [8,11,17,18]. Inadequate vascular function was also reflected by the increased hypoxia observed in tumors treated with soluble DLL4 [17]. Two major changes following DLL4/NOTCH1 blockade might contribute to the defective function of the tumor vasculature: impairment of lumen formation and promotion of a chaotic vascular network. Impaired lumen formation was described in the aortas of Dll4^+/- ^embryos [2]. Antagonizing DLL4/NOTCH1 also caused a morphological change of endothelial sprouts formed in an *in vitro *3D culture system, with the luminal space being absent or greatly reduced [8]. Conceivably, a similar change in tumor vessels may lead to a reduction in vessel lumen size that is incompatible with the passage of red blood cells. Indeed, tumor vascular histology and imaging studies have revealed a shift to smaller vessel calibers and inefficient blood flow in anti-DLL4 treated tumors [20] (unpublished observations). Since inhibition of DLL4 leads to excessive branching of tumor vessels, the other plausible cause of inefficient blood flow could be the manifestation of a chaotic vascular network lacking a functional hierarchy. DLL4 blockade could worsen the already impaired vascular communication in the tumor microvascular network and lead to exacerbated functional shunting, a suspected primary cause of dysfunctional microcirculation and local hypoxia in cancer [21].

In addition to tumor studies using anti-DLL4 as a single agent, additive anti-tumor activity was observed in combination with anti-VEGF therapy in a majority of tested tumor models. Since angiogenic sprouting after DLL4 blockade remains a VEGF-dependent process [8], DLL4 inhibition may increase the dependency of the tumor microvasculature on a VEGF-mediated survival signal. In anti-Dll4 treated neonatal mouse retinas, there was a defect in arteriogenesis with a complete absence of pericyte coverage of the retinal vessels [8]. Soluble DLL4 was also able to reduce the recruitment of pericytes in a murine xenograft tumor model [18]. Therefore, DLL4 blockade may impair the remodeling of the tumor vasculature to become more mature and stable, resulting in increased vulnerability of tumor vessels to VEGF blockade. In tumors targeted by DLL4/NOTCH1 inhibition, the hyperproliferative state of endothelial cells, together with the reduced protection of the tumor endothelium by supporting cells, may render the tumor vasculature more susceptible to agents that selectively target proliferating cells. Indeed, anti-DLL4 in combination with chemotherapy shows enhanced anti-tumor activity in preclinical tumor models despite the concern that reduced perfusion of tumor vessels might interfere with the delivery of therapeutic agents [15,16].

Existing data support the endothelial cell-autonomous role for DLL4/NOTCH1 signaling in restricting the angiogenic response [22]. The NOTCH pathway has been implicated in a variety of human cancers in connection with the genetic alterations and epigenetic events that lead to either constitutive NOTCH activation or sensitized response to ligand-induced activation [12]. Interestingly, a recent study has suggested that DLL4 blockade may reduce tumor-initiating cell frequency in certain xenograft models [15,16]. At present, however, the mechanism underlying DLL4-mediated tumor initiation and/or progression remains unclear.

## Safety challenges

Targeting a key step in the generation of amyloidogenic peptides and the proteolytic activation of NOTCH signaling, a number of GSIs are currently in preclinical and clinical development for indications ranging from Alzheimer disease to T-cell acute lymphoblastic leukemia (T-ALL)[23]. A major hurdle to the therapeutic development of GSIs has been the on-target toxicity in the gastrointestinal tract. Inhibition of NOTCH signaling results in goblet cell metaplasia due to the skewed differentiation of epithelial cells in the intestinal crypts away from an enterocyte fate and towards that of a secretory goblet cell [24]. Development of goblet cell metaplasia requires simultaneous disruption of both *Notch1 *and *Notch2 *[25]. Because GSIs indiscriminately block all NOTCH receptors, targeted inhibition of individual receptors might help to alleviate the observed gut toxicity. Using paralogue-specific anti-NOTCH antibodies, Wu et al recently showed that selective NOTCH1 inhibition resulted in only mild goblet cell metaplasia, representing an important progress in overcoming the toxicity associated with pan-Notch inhibitors [11]. Recently, Radtke's group identified DLL1 and DLL4 as the key physiological ligands that mediate NOTCH signaling in the mouse intestinal epithelium [26]. Intestine-specific inactivation of either *Dll4 *or *Jag1 *had no obvious phenotype, while loss of *Dll1 *resulted in a moderate increase in goblet cell numbers. *Dll1-Dll4 *double knockout mice developed intestinal abnormalities to a degree similar to *Notch1-Notch2 *compound mutant mice [26]. These findings are consistent with earlier work showing that neutralizing anti-DLL4 antibodies had no discernable impact on goblet cell differentiation and/or the proliferation of crypt progenitor cells [8].

While selective inhibition of DLL4 apparently avoids the gut toxicity that plagued the therapeutic application of GSIs, other safety concerns have been raised [27]. Administration of a DLL4-specific neutralizing antibody caused a rapid and significant change in mouse liver gene expression, including the upregulation of endothelium- specific genes as well as genes implicated in proliferation and cell cycle regulation. More importantly, mice, rats and cynomolgus monkeys exposed to anti-DLL4 antibody developed histopathological changes in the liver, including profound sinusoidal dilation and centrilobular hepatocyte atrophy [27]. These changes are believed to be a class effect of inhibiting DLL4 signaling through NOTCH1, since similar changes were also observed when DLL4/NOTCH1 signaling was inhibited by agents targeting NOTCH1 or γ-secretase. Along with the histological findings, hepatic clinical pathology changes were observed in treated animals. These findings suggest that DLL4 signaling is essential for maintaining the structural and functional integrity of the liver sinusoidal endothelium as well as hepatocyte homeostasis. Since it has been documented that some chemotherapy agents could cause liver sinusoidal dilation or other types of liver damage [28], extra precaution should be taken when combination therapy is administered.

Besides the liver clinical- and histopathology changes observed upon exposure to anti-DLL4 antibody, other concerning safety signals were seen following prolonged treatment. Most strikingly, skin lesions with features of vascular neoplasms were observed in male rats. In addition, rare tumors with similar features were identified in heart and lung [27]. Among the species tested, which included mice, rats and monkeys, the proliferative vascular lesions were reported only in rats after 8 weeks of continuous anti-DLL4 exposure. However, the potential consequences of longer drug exposure in other species remain uncertain. In clinical trials, patients treated with semagacestat (LY450139), a γ-secretase inhibitor, had an increased risk of skin cancer [29]. A recent study employing an elegant genetic model showed that loss of *Notch1 *caused widespread vascular tumors, particularly in the liver, further underscoring the potential safety concerns associated with continuous blockade of NOTCH1 signaling [30].

Accumulating evidence suggests that DLL4-mediated NOTCH signaling is not restricted to the vascular compartment. Recent genetic studies have identified DLL4 expressed by thymic epithelial cells as the essential and nonredundant NOTCH1 ligand responsible for intrathymic T cell development [31,32]. Consistent with DLL4 genetic inactivation, anti-DLL4 treatment resulted in a complete blockade of T cell development coupled with ectopic appearance of immature B cells in the thymus (unpublished observations). In addition, DLL4 together with DLL1 regulate NOTCH signaling in the intestinal crypt [26]. The recognition of a broader role for DLL4-mediated NOTCH signaling raises the concern that on-target toxicity of DLL4 inhibition could extend beyond the endothelium.

While preclinical models have revealed the potential toxicities associated with DLL4/NOTCH1 blockade, safety findings in humans have just begun to emerge from recent clinical trials. DLL4 targeting antibodies, OMP-21M18 and REGN421, have entered clinical development in recent years. In a Phase I study of OMP-21M18, 28% of patients experienced grade III asymptomatic hypertension, a condition that has not been described in preclinical studies [33]. Since hypertension is a known dose-limiting side effect of anti-VEGF therapy [34,35], patients receiving combination therapy of anti-DLL4 and anti-VEGF should be carefully monitored.

## Potential path forward for targeting DLL4/NOTCH1

Preclinical studies have suggested that DLL4/NOTCH1 blockade could augment the efficacy of anti-VEGF in "sensitive" tumors. DLL4/NOTCH1 blockade may also have the potential to enhance the effects of chemotherapy or other targeted therapies in tumors that are either intrinsically less dependent on VEGF or have progressed due to a shift to dependency on other angiogenic pathways. While significant safety concerns have been raised in recent preclinical studies, the full therapeutic potential of targeting DLL4/NOTCH1 should be further explored given its remarkable impact on the tumor vasculature and tumor growth in preclinical models. Lessons learned from the preclinical and clinical development of GSIs may offer some clues for a potential path forward. Since it is generally believed that the outcome of Notch signaling is dependent on context and the degree of activation, it is possible that partial pathway inhibition might ameliorate the toxicities associated with stringent and continuous pathway blockade while maintaining efficacy. Indeed, Cullion et al were able to show that a GSI dosing schedule with drug holidays largely avoided the gut toxicity while maintaining significant efficacy in mouse T-ALL models [36,37]. It will be interesting and exciting to determine if strategies based on the same principle could expand the therapeutic window of selective targeting of DLL4/NOTCH1. The other remarkable observation from Ferrando's group was that dexamethasone protected mice against GSI-induced gut toxicity by shifting differentiation away from goblet cell development [38]. Since the effects of DLL4/NOTCH1 blockade might be partly attributed to enhanced VEGF signaling, anti-VEGF may have the potential to protect against anti-DLL4 mediated toxicity. Interestingly, it has been reported that bevacizumab protects against injury in patients treated with oxaliplatin-based chemotherapy, which is known to cause liver sinusoidal dilation [39]. Finally, it is important to point out that, given the potent effect of DLL4/NOTCH1 blockade and its potential safety implications, future development of anti-DLL4/NOTCH1 therapeutics will require careful monitoring of patient safety until the relevance and/or translatability of observed preclinical toxicity concerns are better understood in the clinical setting.

## Competing interests

The author declares that they have no competing interests.
